# The Tenth International Medical Congress

**Published:** 1890-09

**Authors:** Eric E. Sattler

**Affiliations:** Berlin


### CORRESPONDENCE.

### The Tenth International Medical Congress.

BERLIN, AUGUST 5, 1890.

*Editor Lancet-Clinic:—*

The Tenth International Medical Congress has opened in a blaze of glory. At 11 A. M. yesterday the Circus Renz, in which the general meetings are held, was filled to the utmost extent with a suffocating mass of representatives of all nations. It was a broiling hot day, and the heat was rendered still more intense by the hundreds of gas and electric lights that gave illumination to the interior of the Circus. As can be imagined the acoustic properties of the circular, amphitheatre-like building, used as a circus in winter, were perfectly miserable, and the speakers could be heard but by the few that faced them directly. So the most were content to catch a glimpse of the famous men that spoke, and read their articles later in the transactions. That great pathologist, Virchow, whose name is probably familiar to a greater number of medical men than any other living scientist, presided. His voice was entirely too feeble to be heard at any distance. Secretary Lassar gave an interesting synopsis of the work done by the organization committee, the number of members present, etc. He stated that about 5,000 members had been enrolled, of which 2,500 were Germans, and about an equal number foreigners. He emphasized the fact that the French Government had sent a delegation, and that some of the best French medical *savants* were present. This statement was received with a storm of applause quite prolonged. Considering the feeling that has existed for some time on this Franco-German medical question, it seemed as if the pipe of peace had been permanently smoked by these factions.

A very interesting fact which the secretary announced was that the United States was represented by 500 delegates, next to Germany itself the greatest number of representatives—even Great Britain and Ireland being only about 300 strong. About

1,000 ladies, wives of members, were also enrolled. The printing of the titles of papers announced would fill a book of seventy pages, and the number of titles reach almost 1,000. A number of deputed personages of Berlin, and various prominent representatives of other countries, in turn addressed the audience both in words of welcome and thanks. Hamilton, of Washington, the Secretary of the Ninth International Medical Congress, spoke for America, and Sir James Paget for England. The announcement of Sir James Paget's name was greeted with prolonged, enthusiastic applause which quite assumed the form of an ovation when Sir James arose to address the meeting.

In following the proceedings and addresses of the congress, in the midst of this great concourse of men, the fact that these international medical meetings were fast becoming unwieldy and unmanageable could not but impress itself on me. It seems that in some future time ways and means must be found to obviate this difficulty. The only solution I can see is in international *sectional* congresses, *i. e.*, an international congress for internal medicine and pathology; for surgery, otology, laryngology and rhinology, etc.; or that, if a general international congress is held, a certain specified number of delegates only from each country be admitted.

The section meetings are held in the "Landes-Austellung," a picture gallery some twenty minutes' walk from the Circus Renz. The sections of Otology and of Rhinology and Laryngology are well filled. It seems a pity that these two sections have not been made one, as no two departments are more closely united by inseparable links of pathology. There are about 125–150 members in the section of Rhinology and Laryngology. Among them are such prominent men as Lennox Browne, of London; McBride, of Edinburgh; Butlin and Semon, of London; Fraenkel, Schech, Gottstein, Krause, Stoerk, Schrotter, Chiari, Hartmann, of Germany and Austria; Massei, of Italy; Gongenheim and Moure, of France; Bosworth, Lefferts, Daly, Casselberry, Gleitsman, Jarvis, of America.

The President of the section, Professor B. Fraenkel, delivered the introductory essay on "Laryngology Since the International

Congress in 1887." He dwelt upon the importance of laryngology, its ever-increasing field of usefulness, and incidentally referred to the stimulus that had been given to the study of carcinoma of the larynx in the last two years; and while not mentioning names, gave Sir Morell Mackenzie a sharp cut and severe criticism. This reference was credited with applause and shouts of "bravo." From what nationality the applause originated I am not able to state positively, but can well imagine. In my next letter I will come to speak more fully of some of the interesting subjects discussed in this section—subjects that will interest the general practitioner as well as the specialist.

As I stated before, America sends the second largest delegation to the congress. I have taken the trouble to carefully go over the list of the 5,000 members of the Congress to see what portion of our country was best represented. I find that the section west of the Alleghanies send about ten more representatives than that east of these mountains. When we consider that New York, Philadelphia, Boston, Baltimore and Brooklyn are east of the Alleghanies, I think it makes a splendid showing for our western colleagues. I make this comparison especially on account of the criticism in various journals about the appointment of the committee for America entirely from members of our profession east of the Alleghanies. Not quite one-half of this committee are present at the congress.

The social part of the programme has been on a great scale. A reception at the town hall, a ball, section dinners, excursions to various suburbs and places of interest, social reunions in Kroll's Gardens, etc., are sufficient in number to vary the protracted busines and sectional meetings.

Berlin is certainly one of the best situated cities for holding a general convention, having in the last fifteen years made strides progressive in nature as no other city in the world.

At the general meeting to-day Rome was chosen as the place for holding the next International Convention.

ERIC E. SATTLER.

